# Novel Drugs Targeting the Epidermal Growth Factor Receptor and Its Downstream Pathways in the Treatment of Colorectal Cancer: A Systematic Review

**DOI:** 10.1155/2012/387172

**Published:** 2012-10-14

**Authors:** Amartej Merla, Sanjay Goel

**Affiliations:** ^1^Department of Medicine, Montefiore Medical Center, 111 210th Street, Bronx, NY 10467, USA; ^2^Department of Medical Oncology, Montefiore Medical Center, Albert Einstein College of Medicine, 1825 Eastchester Road, Bronx, NY 10461, USA

## Abstract

Colorectal cancer is the second most common malignancy among men and women in the United States, and the 5-year survival rate remains poor despite recent advances in chemotherapy and targeted agents. The mainstay of therapy for advanced disease remains the cytotoxic chemotherapy including 5-FU, irinotecan, and oxaliplatin. The USFDA approval and introduction of targeted therapies, including cetuximab and panitumumab (monoclonal antibodies targeting the epidermal growth factor receptor (EGFR)) and bevacizumab (monoclonal antibody targeting the vascular epithelial growth factor (VEGF)), has improved the median survival of patients with metastatic colorectal cancer to around 24 months. Clearly, better and more efficacious drugs are needed, and target-specific agents remain the future of cancer treatment. On this front, rapid advances are being made, which are likely to change the future of the management of metastatic colorectal cancer. However, absence of specific biomarkers for the use of targeted agents, in the subset of population who will benefit from the treatment, remains a major drawback. In this paper, we review agents that are in phases 1 and 2 clinical development, specifically targeting the EGFR and its subsequent downstream pathways.

## 1. Introduction

 Colorectal cancer (CRC) is the second most common cause of cancer-related deaths in the United States. The American Cancer Society estimates that in 2011 around 141,210 Americans were diagnosed with CRC of which 49,380 succumbed from the disease [1]. Over the past several decades, the incidence and mortality of CRC have declined. The treatment for colorectal cancer has transitioned from single agent chemotherapy to combination cytotoxic therapies and target-specific agents. Fluoropyrimidines, irinotecan, and oxaliplatin are the main drugs for cytotoxic chemotherapy. The standard of treatment for metastatic CRC (mCRC) is FOLFOX (5 fluorouracil, leucovorin, and oxaliplatin) or FOLFORI (5 fluorouracil, leucovorin, and irinotecan). Bevacizumab, cetuximab, and panitumumab are the target-specific agents approved by FDA for the treatment of colorectal cancer [2, 3]. The present combination of cytotoxic chemotherapies and the addition of target-specific agents have increased the overall survival of metastatic colon cancer to around 24 months [4–7]. 

## 2. EGFR Signaling Pathway

**Figure 1:**
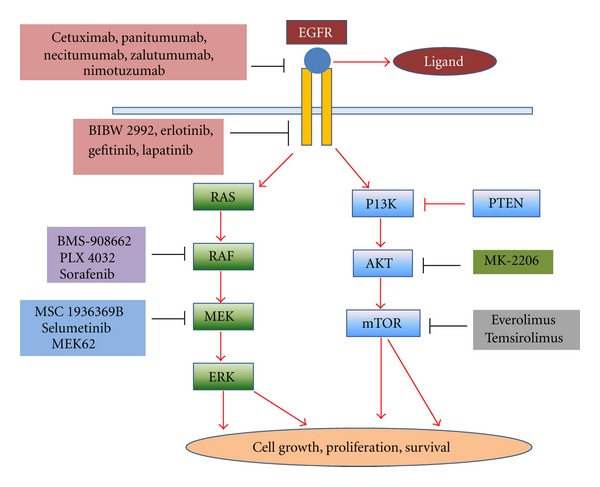
Schematic diagram showing various drugs acting on EGFR and its subsequent pathways. MEK: MAPK (mitogen-activated protein kinase) kinases/extracellular-signal-regulated kinases, ERK: extracellular-signal-related kinase; PTEN: phosphatase and tensin homolog, mTOR: mammalian target of rapamycin.

Human tumors are rich in growth factors and their receptors. Among the mostly widely studied is the EGF receptor family [8, 9]. The EGFR gets activated after a ligand binding, which in turn activates 2 pathways, the RAS-RAF-MEK-ERK pathway and the PI3-AKT-mTOR pathway. Drugs which act on this receptor can be classified into 3 subcategories (Figure 1):

- drugs that inhibit the extracellular domain,
- drugs inhibiting RAS-RAF-MEK-ERK pathway,
- drugs inhibiting PI3-AKT-mTOR pathway.

Cetuximab (an IgG1 monoclonal antibody) and panitumumab (fully human IgG2 monoclonal antibody) are the only monoclonal antibodies against EGFR that are approved for treatment of metastatic CRC. Only small subsets of patients show clinical benefit to cetuximab and panitumumab. Patients who have KRAS mutation are resistant to cetuximab [6]. Mutations of KRAS lead to activation of RAS-RAF-MEK pathway which renders an inhibition in the receptor further upstream fairly ineffective. Recently BRAF mutation and loss of PTEN were also attributed to resistance to cetuximab and panitumumab therapy [10–12]. KRAS mutations are seen in 40–50% of CRC, while BRAF mutations are seen in 10% of colorectal cancer. The best response to cetuximab and panitumumab appears to be in patients who have a combination of wild-type KRAS, BRAF, and PIK3CA and express the phosphatase and tensin homolog (PTEN) protein [12–14]. PTEN is a tumor suppressor protein that inhibits the PI3/AKT pathway, and loss of this protein will activate this pathway leading to tumor progression. 

## 3. Novel Drugs in Phase 2 Clinical Development

### 3.1. Inhibitors of EGFR/Drugs Acting on Extracellular Ligand Binding Domain

**(1) BIBW 2992/Afatinib**

Afatinib is a highly selective inhibitor of EGFR and HER2 currently undergoing phase 1 trials for various solid tumors [15, 16]. It is a second-generation EGFR-TKI (tyrosine kinase inhibitor) and has shown promising results in advanced non-small-cell lung cancer (NSCLC) [17]. The LUX-lung clinical trial program was a phase 2b/3 randomized, double-blinded trial which showed promising results in NSCLC with a statistically significant increase in median PFS by 2 months. The main toxicities included diarrhea and skin rash which in most cases were managed by dose interruption or reduction [18]. There are currently phase 2 trials for BIBW2992 in metastatic (m) CRC. A phase 2 trial has been conducted by alternating BIBF 1120, a potent angiokinase inhibitor, and afatinib in 46 patients who already received several lines of chemotherapy. Seven patients remained progression-free after 16 weeks. Most of the patients tolerated the drugs with manageable toxicity [19]. Currently a phase 2 trial is ongoing (National Clinical Trial (NCT) 01152437), which compares the efficacy of cetuximab and afatinib. Patients with mCRC who had progressed on oxaliplatin or irinotecan and have not received any anti-EGFR therapy are eligible for the study. Patients with wild-type KRAS are randomized to cetuximab or afatinib, while those with mutant KRAS will receive afatinib.

**(2) Necitumumab/IMC-11F8**

Necitumumab is a fully human IgG1 monoclonal antibody against EGFR [20]. Preclinical trials have shown that the efficacy of necitumumab can be compared to cetuximab [21]. Currently this drug is undergoing phase 3 trials for NSCLC. A phase 2 trial in CRC has been completed and has not been published. This study compared patients with mCRC either randomized to mFOLFOX-6 regimen or combination of necitumumab and mFOLFOX-6 (NCT 00835185). Preliminary data presented at 2008 annual American Society for Clinical Oncology (ASCO) meeting suggests that the combination is well tolerated with notable benefit in tumor activity [22, 23]. The median PFS (progression-free survival) or OS (overall survival) has not yet been reported.

**(3) Erlotinib**

Erlotinib is an oral, reversible EGFR-TKI. Erlotinib has been approved for use in metastatic NSCLC in patients who have stable disease after 4–6 cycles of first-line chemotherapy, as a maintenance therapy [24]. Several trials have shown its efficacy as a first-line agent compared to standard chemotherapy in EGFR mutant patients with NSCLC [25]. Erlotinib has also been approved for its use in pancreatic cancer [26]. Erlotinib is being tested in a number of phase 2/3 trials in advanced CRC [27–30] (Tables 1 and 2).

**(4) Gefitinib**

Gefitinib is a reversible EGFR-TKI used in the treatment of locally advanced or metastatic NSCLC with EGFR mutation, approved in Europe [31] and not in the United States. It is currently being tested for mCRC in phase 2 clinical trials. A number of trials are under progress and a couple of them have been published. A phase 2 trial combining capecitabine and gefitinib as second-line therapy in advanced CRC showed no added efficacy and resulted in severe skin toxicities [32]. A trial involving combination of raltitrexed and gefitinib in one arm and raltitrexed alone has been tested on 76 patients as a second-line chemotherapy in advanced mCRC. This combination was well tolerated, but there was no difference in PFS between the arms [33]. The combination of FOLFOX-4 and gefitinib (IFOX) has been tested on previously untreated patients with mCRC. This trial showed increased efficacy in patients treated with IFOX when compared to FOLFOX-4 alone in a similar setting. The median OS was 20.5 months. Grade 3-4 diarrhea was reported in 67% patients, and Grade 3-4 neutropenia was reported in 60% of the patients, which is higher than FOLFOX-4 alone. The limitations of this study were that it does not have a control arm to compare efficacy [34]. Conflicting results were published on the efficacy of the combination of gefitinib and FOLFOX as first-line chemotherapy [35]. A multicenter phase 2 trial did not show any added benefit with gefitinib as a first-line agent [36]. A phase 2 randomized multicenter trial compared FOLFIRI to a combination of FOLFIRI and gefitinib and showed no overall benefit but has shown significant increase in toxicities [37]. All the above-mentioned phase 2 trials with gefitinib as first line or maintenance therapy in mCRC showed increased toxicities of gefitinib. Various phase 2 trials are being conducted on gefitinib in mCRC, and results are available from few trials [38, 39] (Table 3).

### 3.2. Novel Drugs in Phase 1 Clinical Development That Act on EGFR

**(1) Zalutumumab**

This is a novel human IgG1 monoclonal antibody against EGFR. It is currently being tested in phase 2 trials for squamous cell carcinoma of head and neck, and the results appear to be encouraging [40]. A phase 1 study on zalutumumab and irinotecan in mCRC after failed irinotecan and cetuximab, presented at 2009 annual ASCO meeting, showed stable disease warranting further studies [41].

**(2) Lapatinib**

This is an oral drug and a dual inhibitor of EGFR-TKI and HER2. It is currently undergoing phase 4 trials in HER 2 positive advanced metastatic breast cancer. A phase 1 trial in advanced solid tumors that includes mCRC has been published. It shows efficacy towards certain types of solid tumors [42]. Its efficacy and toxicities in a phase 2 CRC-specific studies are yet to be tested.

**(3) Nimotuzumab**

An IgG1 human monoclonal antibody with efficacy in head and neck cancer is undergoing phase 2 clinical trials in China. It is being tested in combination with irinotecan, as a second-line agent for mCRC having wild-type KRAS (NCT 00972465).

### 3.3. Drugs Inhibiting RAS-RAF-MEK-ERK Pathway

Various targeted therapies that inhibit the components of this pathway are in clinical trials. The RAS-RAF-MEK-ERK which is involved in regulation of cell cycle is activated in many cancers due to mutations in BRAF or RAS genes [43, 44]. This is a system of protein kinases where a member of RAF (A-RAF, B-RAF, and C-RAF) kinase phosphorylates and activates the MEK kinases (MEK1 and 2) which phosphorylates to further activate ERK1 and 2, which in turn phosphorylate to activate a number of substrates that are key components in cell cycle regulation. This pathway is coordinated by the binding of active RAS (HRAS, NRAS, and KRAS) to RAF [45].

**(1) BMS-908662**

This drug is a RAF inhibitor and currently undergoing phase 1/2 trials in mCRC alone or in combination with cetuximab in patients with mutated KRAS or BRAF. The study is recently completed, and the results are pending (NCT 01086267).

**(2) MSC 1936369B**

This is a MEK inhibitor that is tested in phase 2 CRC studies. This is being investigated as a second-line agent in combination with FOLFORI in patients whose tumors harbor a KRAS mutation (NCT01085331).

**(3) Selumetinib/AZD6244**

This is an orally available drug and a selective inhibitor of MEK1/MEK2. The drug is currently undergoing phase 2 trials in colon cancer, melanoma, hepatocellular carcinoma, and biliary cancer [46]. A phase 2 randomized multicenter study comparing oral capecitabine and selumetinib as a second-line or third-line chemotherapy in advanced CRC demonstrated good tolerability and equal efficacy. The median PFS between both arms was not significantly different. The most common side effects reported in selumetinib are dermatitis, diarrhea, and asthenia [47]. This drug is currently being tested in many phase 2 trials in mCRC (Table 4).

**(4) PLX 4032/Vemurafenib**

This drug is an oral selective inhibitor of oncogenic V600E mutant BRAF kinase. This mutation is found in up to 50% of patients with malignant melanoma but also found in a small subset of colorectal cancer patients. A phase 1 study is investigating this drug in mCRC (NCT00405587). Several studies have shown promising results in malignant melanoma leading to its approval by the USFDA for this indication [48]. A phase 1 trial evaluating the role of PLX 4032 in patients with advanced CRC with mutant BRAF was presented at 2010 annual ASCO meeting. The results were modest when compared to melanoma but clearly indicate that targeting mutant BRAF is a therapeutic option in colorectal cancer [49]. A preclinical study presented at 2012 annual Gastrointestinal Cancers Symposium, combining PLX4032 which is a BRAF inhibitor, and MEK inhibitor suggested synergistic effect of combined pharmacological blockade of the entire pathway [50].

**(5) Sorafenib**

Sorafenib is an oral RAF and multitargeted TKI. Though initially discovered as potent RAF inhibitor, it is now know that it also inhibits vascular endothelial growth factor receptor (VEGFR) and platelet-derived growth factor receptor (PDGFR). Its inhibiting action on RAF is in the order of RAF > wild-type BRAF > oncogenic B-RAF V600E [51]. Sorafenib is now FDA approved for its use in advanced renal cell carcinoma and unresectable hepatocellular carcinoma. Its efficacy is now being tested in various solid tumors, and phase 2 trials are in progress for CRC. A phase 2 study was conducted in Saudi Arabia on 35 patients with mCRC who progressed on first-line chemotherapy. Patients were randomized to combination of cetuximab and sorafenib or cetuximab alone. Results show that the combination arm had higher partial response rate and an improved PFS especially in patients with wild-type KRAS status as compared to those with a mutation [52]. Several phase 2 trials of sorafenib in CRC are ongoing (Table 5).

**(6) MEK 62/ARRY-438162**

This is a potent selective inhibitor of MEK1/MEK2. This is currently in phases 1 and 2 trials for many advanced solid tumors with KRAS, NRAS, and BRAF mutations (NCT01363232, NCT01337765, NCT01363232). A phase 1 multicenter study on the safety of MEK 62 on biliary cancer has been recently presented at 2012 Gastrointestinal Cancers Symposium. The same study is being expanded to include patients with CRC with KRAS and BRAF mutations [53].

### 3.4. Drugs Inhibiting PI3 K-Akt-mTOR Pathway

This pathway is important for cell growth and survival. Abnormal activation of this pathway predisposes to development of many cancers, and genetic mutations in this pathway are common in many malignancies. Hence this pathway has gained importance in recent years as a target for drug development. Inhibitors of this pathway are in phases 1 and 2 clinical trials. PI3KCA mutations are seen in 25% of colorectal cancers [54]. Mutations in PTEN, AKT2, and PDK1 are also implicated in CRC [55]. PTEN inhibits this pathway, and loss of PTEN or mutation leads to increased cell proliferation and decreased apoptosis [56].

**(1) MK-2206**

This is an oral AKT inhibitor. A phase 2 trial of MK 2206 and AZD6244 in advanced CRC is recruiting patients. This trial aims at simultaneously blocking the PI3 K-AKT and RAS-MAPK pathways (NCT01333475). The first human phase 1 trial of MK 2206 in advanced solid tumors was recently published. The drug was well tolerated, showing AKT blockade. Toxicities reported were skin rash, nausea, purities, hyperglycemia, and diarrhea [57].

**(2) Everolimus**

This is an oral derivative of rapamycin and approved by the FDA for management of patients with advanced renal cell carcinoma who have failed one prior line of therapy [58]. Rapamycin is an mTOR inhibitor approved in many countries to prevent rejection of solid organ transplants. A nonrandomized phase 2 trial on fifty metastatic colorectal cancer patients who failed first-line chemotherapy and cetuximab or panitumumab was enrolled in the study. Patients received bevacizumab every 2 weeks and daily everolimus. The drug was well tolerated. The median OS was 8.1 months showing only modest clinical efficacy [59]. There are many phase 2 trials under investigations in advanced CRC (Table 6).

**(3) Temsirolimus**

This is an intravenous mTOR inhibitor approved by the USFDA for advanced renal cell carcinoma [60]. It is currently undergoing phase 4 clinical trials in mantle cell lymphoma. This drug is currently in phases 1 and 2 trials for advanced CRC. This drug has been evaluated in patients with KRAS mutation whose cancer was irinotecan resistant, and the results are yet to be reported. (NCT00827684). Rare but serious side effects include interstitial lung disease, acute renal failure, and bowel perforation.

## 4. Discussion

 Despite recent advances in our knowledge at the molecular level of colorectal cancer, the prognosis still remains poor. The 5-year survival rate in a tertiary oncology center in the United States is around 10% for advanced CRC [61]. Though the 5-year survival rate has not changed with the recent advances, the 2-year survival rate significantly improved for metastatic colorectal cancer, around 40 percent in recent years compared to 20 percent a decade ago [62]. Chemotherapy still remains the mainstay of treatment [63]. Combining traditional chemotherapy with targeted therapies has shown benefit in several studies [64–66]. With this idea in mind and with preclinical data of the efficacy of the combination of anti-EGFR and anti-VEGF therapy, clinical trials were launched. However, the activity observed in preclinical studies did not hold its ground once the clinical results were announced. In CAIRO2 (capecitabine, irinotecan, and oxaliplatin in advanced colorectal cancer) and PACCE (panitumumab advanced colorectal cancer evaluation study) trials, the addition of anti-EGFR antibody to a combination of chemotherapy and bevacizumab significantly reduced the PFS [67, 68]. Targeted drug therapy is a rapidly emerging field, but lack of specific biomarkers to channelize the treatment only to the subset of patients who get benefit from it still remains unsolved. 

 The resistance to cetuximab and panitumumab in patients with KRAS mutation is well known. It is now a standard of care to evaluate for KRAS mutation in metastatic colorectal cancer [13, 69–72]. While KRAS mutation is an excellent well-documented marker of exclusion, it is not, however, a reliable marker of inclusion. In spite of excluding the patients with a KRAS mutation in their cancer from receiving anti-EGFR therapy, the response rates in the patients with WT KRAS are of the order of 17%–60% [6, 13, 69, 70, 73–75]. More importantly, there appears to be a negative outcome when patients with a KRAS mutation are treated with the anti-EGFR drugs [76, 77]. This suggests that there are other potential markers of resistance [78]. This search for other biomarkers of resistance has mainly revolved around the mitogen-activated protein kinase (MAPK) pathway and the PI3 K-AKT-mTOR-PTEN pathway.

 Some studies indicate that BRAF mutations (around 10% in CRC), which are further downstream of the KRAS gene, are markers of resistance to cetuximab or panitumumab. Further studies have now indicated that BRAF is a better prognostic rather than a predictive biomarker [10, 79–84]. Patients with this mutation have poorer prognosis and appear to respond less robustly to the anti-EGFR antibodies [10]. The routine testing of BRAF has still not found its place in clinical practice. As regards the PI3 K-PTEN pathway, multiple components of the pathway have been implicated in its aberration; leading to its overactivity including mutations in PIK3CA, PTEN, PIK3R1 (regulatory subunit of the PIK3CA gene), p85*α* [85], and AKT1. Furthermore, loss of PTEN expression in CRC has been shown to be mediated by promoter hypermethylation [86, 87]. 

 A number of studies indicate that loss of PTEN is associated with poorer outcomes in metastatic CRC and a less robust response when therapy is indicated with the anti-EGFR drugs. Our group was one of the first to demonstrate *in vitro* that mutations in the PIK3CA gene and loss of PTEN expression predicted for resistance to cetuximab in a panel of CRC cell lines [88]. Since then, several clinical studies have been reported, with conflicting results; with some reports suggesting a predictive role of this pathway and others refuting this finding [11, 89–96]. Notably, a recent report suggested that mutations in exon 20 of PIK3CA specifically predicted for resistance to cetuximab [93]. When initially approved, the use of cetuximab was restricted to patients whose tumors “over expressed EGFR” as documented by IHC staining; however, subsequently this restriction was removed when clinical evidence did not support this approach. Baseline EGFR in determining response to cetuximab remains controversial with studies differing in their results [96, 97]. 

 As further data regarding the role of the PI3 K pathway evolves, there is the potential to markedly improving the clinical benefit rate and excluding almost 60–70% of patients from the use of anti-EGFR therapy. While we continue to refine the use of drugs already available, there is clearly a need for newer drug approaches that may be useful in this deadly cancer. The drugs mentioned in this paper offer a beacon of hope on the horizon that approaches targeting the EGFR and its downstream pathways are abundant. The key question is going to be whether the further development of these drugs should be biomarker driven or should it be tested in all comers. There are risks and benefits to both of these approaches. The need for further biomarkers in both clinical practice and the process of drug development is urgent to enable clinicians to predict responses to different targeted therapies. Many targeted drugs are in phase 2 and 3 trials, the results are being waited to incorporate them in the treatment of colorectal cancer. 

## Figures and Tables

**Table 1:** Phase 2 trials of erlotinib in colorectal cancer.

Study	Status	Results	NCT identifier
Erlotinib, capecitabine, and oxaliplatin	Completed	The arm with erlotinib showed higher response rate and PFS	NCT 00123851
Erlotinib alternating with chemotherapy for second-line treatment	Unknown	Pending	NCT00642746
Bevacizumab and erlotinib in combination with FOLFOX	Completed	High number of withdrawal due to toxicities limiting conclusion on efficacy	NCT00116506
Capecitabine in combination with erlotinib	Terminated	14 patients enrolled, severe toxicities median survival 76 weeks	NCT00459901
Dual epidermal growth factor inhibition with erlotinib and panitumumab with and without chemotherapy	Recruiting	Pending	NCT00940316
Erlotinib in treating patients with recurrent CRC	Completed	Pending	NCT00032110
Dual inhibition of EGFR signaling using cetuximab and erlotinib	Active, not recruiting	Pending	NCT00784667
Intermittent versus continuous Tarceva study	Completed	Pending	NCT01243047
Erlotinib and combination chemotherapy in treating mCRC	Completed	Pending	NCT0049101
Bevacizumab in combination with Xelox and Tarceva	Active, not recruiting	Pending	NCT01135498
Erlotinib in treating patients with history of stage 1, 2, or 3 colorectal cancer or adenoma	Recruiting	Pending	NCT00754494

**Table 2:** Phase 3 clinical trials of erlotinib in colorectal cancer.

Study	Number of enrollment	Expected date of completion	NCT identifier
Chemotherapy and Avastin followed by maintenance treatment with Avastin ± Tarceva	240	December 2011	NCT 00598156
Combination chemotherapy and bevacizumab ± erlotinib in unresectable mCRC	640	Unknown	NCT00265824

**Table 3:** Phase 2 clinical trials of Gefitinib in colorectal cancer.

Study	Status	Results	NCT identifier
IRESSA + Xeloda after failure of first-line chemotherapy	Completed	Pending	NCT 00242788
Gefitinib and combination chemotherapy in advanced or recurrent CRC	Completed	Pending	NCT 00052585
Oxaliplatin ± gefitinib in metastatic or locally recurrent CRC	Completed	Gefitinib and oxaliplatin combination is ineffective in advanced CRC	NCT 00026299
Gefitinib in treating patients with mCRC as a single agent	Completed	Gefitinib as a single agent is ineffective in advanced CRC	NCT 00025350
ZD 1839 in treating patients with advanced CRC that has not responded to chemotherapy	Completed	Pending	NCT 00030524

**Table 4:** Phase 2 clinical trials of selumetinib in colorectal cancer.

Study	Status	Results	NCT identifier
MK2206 and AZD6244 in patients with advanced colorectal cancer	Recruiting	Pending	NCT 01333475
Phase 2 efficacy study of AZD6244 in colorectal cancer	Completed	Pending	NCT00514761
Selumetinib + irinotecan as 2nd-line patients with KRAS and BRAF mutation	Recruiting	Pending	NCT01116271

**Table 5:** Phase 2 clinical trials of sorafenib in colorectal cancer.

Study	Status	Results	NCT identifier
Sorafenib versus placebo + FOLFOX or FOLFORI in the second-line treatment of colorectal cancer	Recruiting	Pending	NCT00889343
Sorafenib + capecitabine in patients with pretreated advanced CRC	Recruiting	Pending	NCT01290926
BAY 43-9006 plus cetuximab to treat colorectal cancer	Recruiting	Pending	NCT00326495
Sorafenib + FOLFORI in CRC after failure of oxaliplatin therapy	Recruiting	Pending	NCT00839111
Sorafenib + capecitabine in previously treated CRC	Recruiting	Pending	NCT01471353
Sorafenib + irinotecan in metastatic CRC and KRAS mutation	Completed	Pending	NCT00989469
Sorafenib, cetuximab, and irinotecan in treating patients with mCRC	Ongoing, not recruiting	Pending	NCT00134069
Sorafenib and bevacizumab versus single agent bevacizumab	Ongoing, not recruiting	Pending	NCT00826540
External-Beam radiation therapy, capecitabine, and sorafenib in treating patients with locally advanced rectal cancer	Recruiting	Pending	NCT00869570

**Table 6:** Phase 2 clinical trials of everolimus in colorectal cancer.

Study	Status	Results	NCT identifier
Panitumumab, irinotecan, and everolimus as 2nd-line in wild-type KRAS	Recruiting	Pending	NCT01139138
Efficacy and safety of everolimus with advanced CRC who failed prior chemo- and targeted therapy	Completed	Pending	NCT00419159
Irinotecan, everolimus, and cetuximab in metastatic CRC with KRAS mutation	Recruiting	Pending	NCT01387880
RAD001, FOLFOX, and bevacizumab in treatment of colorectal cancer	Recruiting	Pending	NCT01047293
Bevacizumab and everolimus in mCRC as a 2nd-line therapy	Completed	Pending	NCT00597506
Phase 2 trial of RAD001 in refractory colorectal cancer	Completed	Pending	NCT00337545
RAD001 and AV-951 in metastatic CRC	Ongoing, not recruiting	Pending	NCT01058655
Safety study of rapamycin administered before and during radiotherapy to treat rectum cancer	Recruiting	Pending	NCT00409994
